# Human Airway Organoids and Multimodal Imaging-Based Toxicity Evaluation of 1-Nitropyrene

**DOI:** 10.1021/acs.est.3c07195

**Published:** 2024-03-28

**Authors:** Yingyan Zhou, Cun Li, Yanyan Chen, Yifei Yu, Xin Diao, Raymond Chiu, Jiacheng Fang, Yuting Shen, Jianing Wang, Lin Zhu, Jie Zhou, Zongwei Cai

**Affiliations:** † State Key Laboratory of Environmental and Biological Analysis, Hong Kong Baptist University, Hong Kong 999077, China; ‡ Department of Microbiology, School of Clinical Medicine, Li Ka Shing Faculty of Medicine, The University of Hong Kong, Hong Kong 999077, China

**Keywords:** human airway organoids, adult stem cell, multimodal imaging analysis, MALDI-MSI, 1-NP

## Abstract

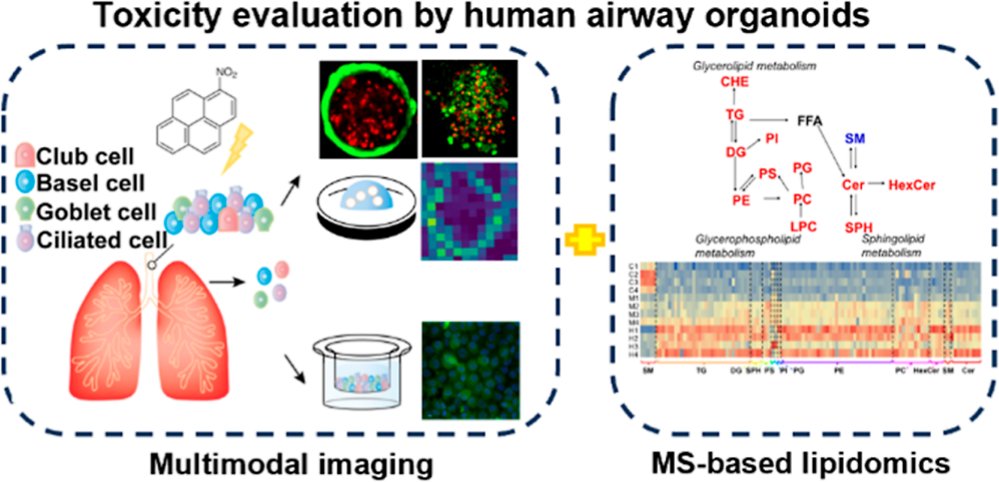

Despite significant advances in understanding the general health impacts of air pollution, the toxic effects of air pollution on cells in the human respiratory tract are still elusive. A robust, biologically relevant in vitro model for recapitulating the physiological response of the human airway is needed to obtain a thorough understanding of the molecular mechanisms of air pollutants. In this study, by using 1-nitropyrene (1-NP) as a proof-of-concept, we demonstrate the effectiveness and reliability of evaluating environmental pollutants in physiologically active human airway organoids. Multimodal imaging tools, including live cell imaging, fluorescence microscopy, and MALDI-mass spectrometry imaging (MSI), were implemented to evaluate the cytotoxicity of 1-NP for airway organoids. In addition, lipidomic alterations upon 1-NP treatment were quantitatively analyzed by nontargeted lipidomics. 1-NP exposure was found to be associated with the overproduction of reactive oxygen species (ROS), and dysregulation of lipid pathways, including the SM-Cer conversion, as well as cardiolipin in our organoids. Compared with that of cell lines, a higher tolerance of 1-NP toxicity was observed in the human airway organoids, which might reflect a more physiologically relevant response in the native airway epithelium. Collectively, we have established a novel system for evaluating and investigating molecular mechanisms of environmental pollutants in the human airways via the combinatory use of human airway organoids, multimodal imaging analysis, and MS-based analyses.

## Abstract 2

“Highly physiologically relevant human airway organoids for assessment of the toxic effects of 1-NP”.

## Introduction

Airborne pollutants, numerous in type and heterogeneous in physicochemical characteristics, have been considered a serious health threat to humans. Air pollution is the most significant environmental cause of death worldwide.^1^ Inhalation is the major route for human exposure to air pollutants.^2^ The respiratory epithelium, particularly the airway epithelium, represents the primary entry and action site for air pollutants.^3^ Countless pieces of evidence, either epidemiological or experimental, have established the causative correlation between air pollution and various respiratory diseases, including allergic airway inflammation, asthma, chronic obstructive pulmonary disease (COPD), and even lung carcinoma.^4,5^ However, although extensive efforts have been made to investigate the toxicology of air pollutants,^6^ the detailed molecular mechanisms of how air pollutants cause respiratory diseases remain poorly understood. One of the hurdles is the lack of a physiologically relevant and readily available in vitro model for mimicking native cells in the human airway.

Human airways are covered with a pseudostratified airway epithelium, with a layer of mucus forming a unique protective air–liquid interface.^7,8^ Ciliated cells with motile cilia are the most abundant cell type in the airway epithelium, along with the other three cell types, i.e., goblet, club, and basal cells.^7,8^ Cell lines derived from cancer cells or tissues are extensively used as in vitro models for biomedical research. However, it is implausible that these homogeneous cell lines can represent native epithelial cells in human organs and tissues. Other in vitro models include human lung explants and primary airway epithelial cells.^8^ Nevertheless, human lung explants are not available on a routine basis.^9^ For primary cells, only sustaining a few passages in vitro is the critical issue limiting their wide applications. Other advanced methods, such as air–liquid interface systems (ALI),^10^ lung-on-a-chip platforms,^11^ and microfluidic single-cell technology^12,13^ have increasingly become popular in environmental toxicology studies. These sophisticated biological systems and technologies offer innovative research methodologies and tools for environmental toxicology. Their applications are limited by the requirement for specialized equipment and the short-term in vitro viability^14^ of primary lung cells (several weeks). Moreover, while lung-on-a-chip systems mimic lung physiology through microengineered designs, such as vacuum-driven cyclical stretching to represent alveolar-capillary barrier movement,^15^ they fall short of fully capturing authentic respiratory dynamics. Similarly, microfluidic single-cell technology, which enables manipulation and analysis at the individual cell level, neglects the complexity of organ architecture and multicellular functions, including tissue organization, cellular communication, and organ-specific processes.

Organoids have become increasingly important for environmental studies as they morphologically and functionally mimic human tissues and organs in vivo.^16^ Human pluripotent stem cell (PSC)-derived alveolar type 2 epithelial cell-like cells were leveraged for toxicological studies.^17^ However, the fetal-like properties intrinsic to PSC-derived organoids and the complexity of establishing these organoids substantially prevent the broad applications of PSC-derived organoids for studying the impact of environmental pollutants on the human airway.^17,18^ Other groups also reported attempts to develop adult airway organoids from adult tissue specimens;^19^ however, the absence of characterizations, including cilia oscillation and cell-type quantitation, obscures the determination of whether those models indeed modeled the human airway epithelium. Progress in stem cell research has allowed the generation of adult stem cell (ASC)-derived organoids.^20^ We have demonstrated the establishment of human airway organoids that recapitulate the human airway epithelium.^9,16,21^ More importantly, the airway organoids could be stably expanded for up to one year, providing a robust and readily accessible in vitro model of the human airway epithelium for environmental studies.^16^

1-Nitropyrene (1-NP), a type of nitrated polycyclic aromatic hydrocarbon (nitro-PAH), is predominantly produced in the exhaust emissions from diesel engines and is commonly found within ambient particulate matter (PM).^22^ This compound is distinguished by its substantial cytotoxic properties and has been classified as a Group 2A carcinogen by the International Agency for Research on Cancer (IARC).^23^ Consequently, 1-NP is acknowledged as a contributor to adverse health effects associated with air pollution and functions as a representative organic pollutant for evaluating environmental contaminants in human respiratory systems. Extensive research, including animal experiments and in vitro cell assays, has indicated the carcinogenesis mechanism of 1-NP, which involves the induction of DNA adduct formation,^24^ the triggering of oxidative stress,^23^ and the disruption of cellular signaling pathways.^25^ These combined effects contribute to the promotion of genetic mutations and cellular transformation. However, the full extent of the impact caused by 1-NP exposure, particularly the perturbation of the lipidome in human airways, is still limited and remains to be investigated.

By examining 1-NP, we demonstrated, as a proof-of-concept, the application of ASC-derived human airway organoids in the toxicological evaluation of air pollutants. Multimodal imaging tools, including live cell and fluorescence imaging and cutting-edge mass spectrometry imaging (MSI) techniques, were used to assess the cytotoxicity of 1-NP to airway organoids. Nontargeted lipidomics was used to quantitatively profile the disruption caused by 1-NP to provide more comprehensive molecular information in addition to multimodal imaging data. The effects of 1-NP on lipidomes were evaluated in airway organoids, and cardiolipin (CL) was found to be affected by 1-NP for the first time.

## Materials and Methods

### Chemicals and Materials

Both 2,5-dihydroxylbenzoic acid (DHB, > 99%) and *N*-naphthylethylenediamine dihydrochloride (NEDC) were purchased from Sigma-Aldrich (St. Louis, MO). Methanol (MeOH), isopropanol (IPA), chloroform, and acetonitrile (ACN) were HPLC-grade (Merck, Darmstadt, Germany). PC (19:0/19:0), LPC (19:0/19:0), TG (15:0/15:0/15:0), Ceramide (Cer) (d18:1–17:0), SM (d18:1/12:0), and sphingosine (d17:1) were acquired from Avanti Polar Lipids. TG (15:0/15:0/15:0) was provided by Sigma-Aldrich (St. Louis, MO).

### Generation of ASC-Derived 3D and 2D Human Airway Organoids

ASC-derived human lung organoids were established using our previously established protocol.^9^ Briefly, organoids were established using lung tissues from patients upon their ethical approval. A single-cell suspension was obtained from lung tissues, embedded in 80% Matrigel (Corning, 356231), and seeded in a 24-well suspension culture plate. Lung organoids were consecutively passaged in the expansion (Exp) medium every 2 weeks with a ratio of 1:3 to 1:5. The proximal differentiation (PD) to generate 3D airway organoids was initiated by transitioning from the Exp medium to the PD medium. Lung organoids were transformed into a 2D monolayer on a transwell plate to derive 2D airway organoids. The beating cilia of organoids were captured using a Nikon Eclipse Ti2 Inverted Microscope System (Movies S1 and S2).

### Immunofluorescence Staining

The differentiated 3D and 2D airway organoids were fixed with 4% PFA, permeabilized with 0.5% Triton X-100, and blocked with a protein block. Cell type-specific antibodies (Table S1) were used to identify four lineages of airway epithelial cells expressed in the airway organoids. DAPI (Thermo Fisher Scientific) and phalloidin-647 (Thermo Fisher Scientific) were applied to counterstain the nuclei and actin filaments, respectively. Confocal images were collected on a Nikon AX confocal laser scanning microscope (CLSM).

### Organoids Exposed to 1-NP

The human airway organoids were developed within an extracellular matrix (ECM), exhibiting an inverted polarity where their apical cell surfaces face inward toward the lumen.^26^ To better model the interaction between environmental toxins and the human airway epithelium in our system, adjustments are required in apically inverted organoids. Therefore, a fragmentation process was implemented to expose the cell surface located inside the organoids to 1-NP.^21^ Briefly, the organoids were slightly fragmented with a Pasteur pipet and mixed with various concentrations of 1-NP (final concentrations: 1, 10, and 50 μM) for 2 h, after which they were then embedded back in Matrigel with 20% medium containing respective concentrations of 1-NP. To examine the dose–response correlation, we elected to use three benchmark concentrations (1, 10, and 50 μM) in our experiments, assessing the impact of 1-NP on airway organoids. These specific concentrations were selected based on previous research findings.^23,27^ A qualitative assessment via microscopic examination, verifying that a majority of organoids have been disrupted, is deemed adequate for evaluating the degree of fragmentation achieved. Representative organoids from each exposure condition were captured with bright-field microscopy by a digital camera on a Nikon C2 Plus confocal microscope.

### Live/Dead Staining

The Calcein-AM and PI working solutions were diluted according to the manufacturer’s instructions (40747ES76, Yeasen). Following this, the airway organoids were stained at 37 °C for 15 min and washed with PBS. The fluorescent intensity of green cells (live) and red cells (dead) was measured using the ImageJ software. The percentage of viable cells was captured by normalizing the signals of the alive cells with those of the total cells.

### Intracellular Reactive Oxygen Species Production Measurement

Intracellular reactive oxygen species (ROS) levels of organoids were measured using 2′,7′-dichlorofluorescein diacetate (DCFDA) (10 μM, 40 min at 37 °C; Thermo Fisher) by fluorescent imaging. After DCFDA probe staining, DNA was stained with 10 μM Hoechst 33342 for 10 min and subsequently washed with PBS three times.

### Acquisition of Lipidomic Profiles

Airway organoids harvested for lipidomic analysis were immediately processed (4 °C, 30 min) with a cell recovery solution (Corning, 354253) to remove the Matrigel. Four biological replicates per treatment condition were prepared, and each replicate used five droplet organoids (approximately 5 × 10^5^ cells). Drawing from a previous study with modifications,^28^ the organoids were added with 750 μL of cold MeOH/H_2_O (4:1, v/v) and ultrasonically disrupted by a homogenizer (Xiaomei, XM-650DT). Subsequently, 450 μL of chloroform was added, and the homogenate was vortexed for 5 min. Then, 450 μL of water was added to promote the phase separation. After equilibrating for 10 min, the mixture was centrifuged at 12,000 rpm for 10 min at 4 °C. The bottom lipid organic phase was dried with an IR concentrator (N-BIOTEK, NB-504CIR). Total protein content was quantified by the BCA assay for normalization. Dried lipophilic cell extracts were reconstituted with 50 μL of ACN/IPA/water (65:30:5, v/v) spiked with 1 μg/mL PC (19:0/19:0), LPC (19:0/19:0), TG (15:0/15:0/15:0), C17 Ceramide (d18:1/17:0), SM (d18:1/12:0), 16:0-d31–18:1 PE, and cholesterol-d7 as internal standards. Quality control (QC) samples were constructed by pooling an equal amount (20 μL) of all of the samples.

LC–MS/MS-based lipidomic analysis was performed on an ultrahigh-performance liquid chromatography (UHPLC) system coupled to an Orbitrap Fusion Lumos Tribrid Mass Spectrometry (MS) system (Thermo Fisher Scientific, USA). The UHPLC system included an ACQUITY UPLC BEH C18 analytical column (2.1 × 100 mm, 1.7 μm). The detailed chromatographic and MS parameters are provided in the Supporting Information (Text S1).

### Sample Preparation for MALDI-MSI

For the sample preparation, we take a previous study as a ref (29), but with two points of modification. First, we introduced an additional centrifugation step to the original protocol, thereby reducing the minimal organoid volume requirement to approximately 20 μL and conserving both time and resources spent on organoid cultivation. Previous work utilized a 96-well plate as a mold, necessitating a minimum filling volume of 100 μL and containing at least 50 μL of organoids.^29^ However, our initial trials revealed a lengthy incubation period for sufficient organoid growth to achieve the required volume. Decreasing the organoid volume within the gelatin block led to sparse distribution in tissue sections and diminished sample efficiency, hindering the throughput of environmental toxicity assessments. This reduction also complicated organoid imaging using MS and reduced signal-to-background ratios. Our method includes a centrifugation step where organoids are embedded in gelatin. Centrifuge tubes are designed with a sharp bottom, allowing the same volume to reach greater heights. Centrifugation helps organoids gather at the bottom of the centrifuge tubes, increasing their density per slice. This reduces the need for excessive slicing, simplifies MS imaging, and preserves the signal strength. Our approach can also employ molds; however, it necessitates the utilization of custom-designed multiwell plates with sharp-bottom features to optimally exploit the advantages of our methodology. Custom molds tend to be costlier, whereas standard centrifuge tubes suffice for the requirements; thus, in this study, we opted for the use of centrifuge tubes for their accessibility and suitability. In summary, our method achieves comparable results to the original molding technique^29^ while saving time and resources in organoid cultivation; Second, adding a chemical fixation process, which stabilizes cellular proteins and preserves cellular morphology, has been demonstrated to be advantageous for maintaining lipid signals during MALDI MSI of individual cells without substantial lipid signal attenuation, as previously reported.^30^ Based on the above improvements, a modified sectioning method was adopted. Briefly, the organoids were removed from the Matrigel, fixed with 4% PFA for 5 min, and washed with PBS, deionized water, and 150 mM ammonium formate, respectively. A warm 15% gelatin solution and a centrifugal step were utilized to obtain a gelatin block with embedded organoids. Slices with 10 μm thickness were obtained by a cryostat (CryoStar NX70, Thermo Fisher), then thawed, mounted onto ITO glass slides, and dried in a vacuum desiccator before matrix deposition. NEDC (7 mg/mL, 90% MeOH) and DHB (20 mg/mL, 100% MeOH) were sprayed using an M5 TM sprayer (HTX Technologies, Chapel Hill, NC). The detailed instrumental parameters can be found in Table S2.

### Mass Spectrometry Imaging

Matrix-assisted laser desorption/ionization MS imaging (MALDI-MSI) was performed on a timsTOF fleX MALDI-2 instrument (Bruker Daltonics, Bremen, Germany). The mass spectra were acquired at a mass range of *m*/*z* 150–1600 by averaging signals from 200 shots at a repetition rate of 10,000 Hz in reflector mode. Laser energy was configured at 45% for the negative ion mode and 55% for the positive ion mode. A 20 μm resolution was achieved in the single mode with a 20 μm raster width. Before the start of each experiment, mass calibration was conducted using the ESI-L Low Concentration Tuning Mix (Agilent Technologies, CA, USA). MALDI-MSI raw data were first reconstructed into ion images and subsequently analyzed in a SCiLS Lab 2021c Premium 3D (SCiLS GmbH, Germany).

## Results and Discussion

### Derivation and Characterization of 3D Airway Organoids and 2D Airway Organoids

**Figure 1:**
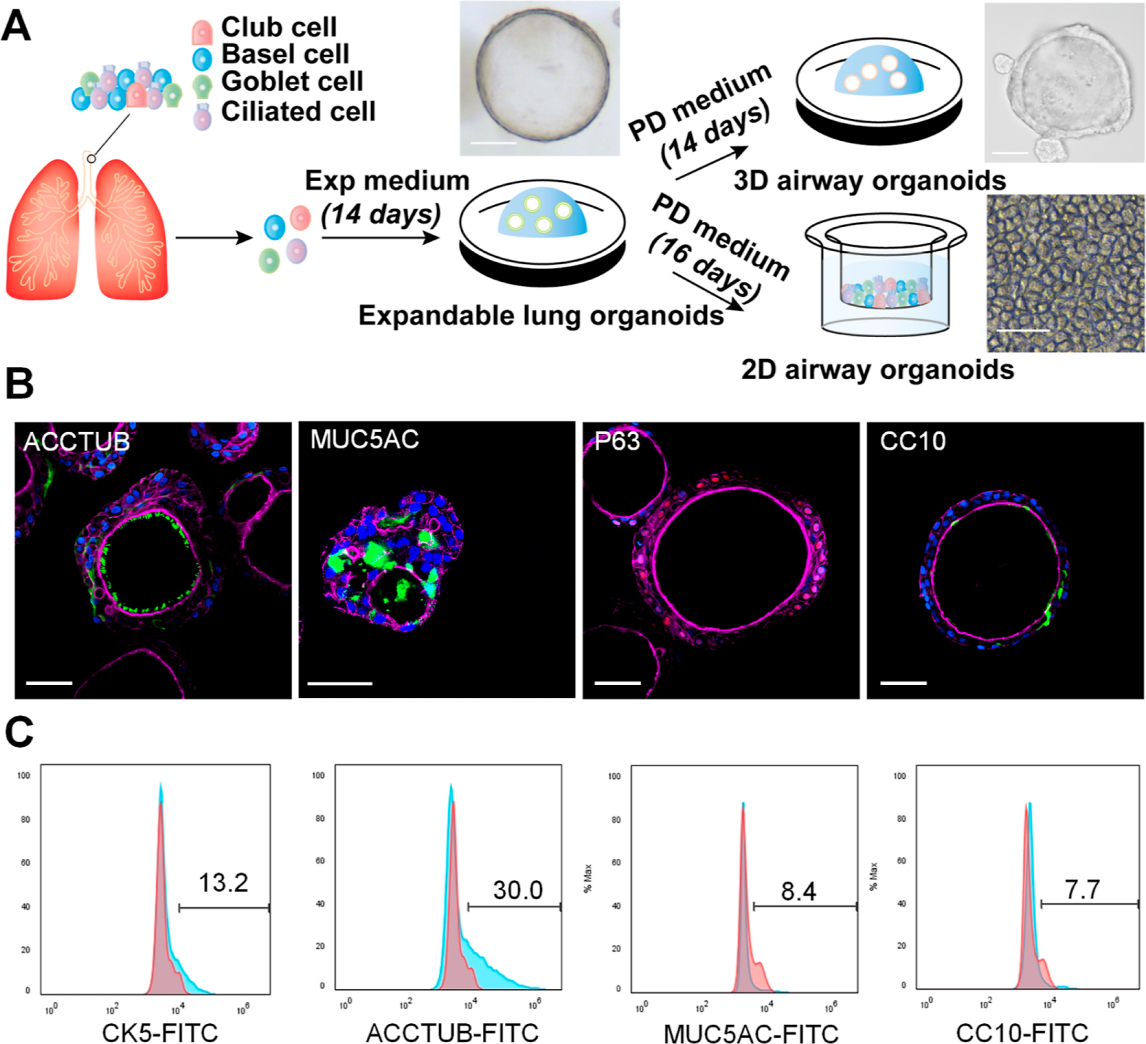
Characterization of differentiated human airway organoids. (A) Schematic diagram illustrates the generation of airway organoids. Photomicrographs display expandable lung organoids, differentiated 3D airway organoids, and 2D airway organoids; scale bar, 100 μm. (B) 3D airway organoids were subjected to immunofluorescence staining to label ACCTUB^+^-ciliated cells (green), MU5AC^+^ goblet cells (green), P63^+^ basal cells (red-orange), and CC10^+^ club cells (green). Nuclei and actin filaments were counterstained with DAPI (blue) and Phalloidin-647 (purple), respectively. Scale bar, 50 μm. (C) Abundance of CK5^+^ basal cells, ACCTUB^+^-ciliated cells, CC10^+^ club cells, and MU5AC^+^ goblet cells in the 3D airway organoids was examined by flow cytometry.

Lung organoids were derived from primary lung tissues with near-perfect efficiency and stably expanded for over one year. A well-defined differentiation protocol enabled us to generate mature airway organoids in both 3D and 2D formats (Figure 1A). Expandable lung organoids displayed a typical morphology of a multicellular sphere with a central lumen and thick wall, whereas 3D airway organoids further differentiated into a more compact structure, and 2D airway organoids established on a transwell membrane became a monolayer of differentiated cells. Beating cilia were discernible in both 3D and 2D airway organoids (Movies S1 and S2). Four major human airway cell types in the differentiated airway organoids were identified by immunofluorescence staining analysis using cell-type-specific markers of ciliated cells (ACCTUB), goblet cells (MUC5AC), basal cells (P63), and club cells (CC10) (Figure 1B). When quantified by flow cytometry, the percentage of each cell type in 3D airway organoids was 30.0, 8.4, 13.2, and 7.7%, respectively (Figure 1C), indicating that airway organoids faithfully simulated the human airway epithelium.^9^ It is worth mentioning that 2D and 3D organoids possess distinct advantages and limitations in the field of environmental toxicology. Our preliminary study showed that both 3D and 2D organoids can serve as viable proof-of-concept models. 2D organoids offer ease of manipulation, characterization, and high-throughput capabilities. However, they only partly represented the physiological 3D structures in vivo. On the other hand, 3D organoids provide a much closer approximation to the physiological and pathological conditions of real tissues, allowing for better simulation of intercellular interactions and microenvironmental influences. Nonetheless, they require higher cultivation and maintenance costs, along with increased technical complexity. Analysis methods and instruments often need customization to accommodate the characteristics of 3D structures. The selection between 3D and 2D organoids depends on the specific research objectives and experimental requirements. If the focus is on intercellular interactions, tissue structure, and cellular behavior under physiological or pathological conditions, then priority should be given to 3D organoids. Conversely, if the research emphasis is on fundamental cellular biology processes, rapid screening, or preliminary validation, 2D organoids may be more suitable.

### Toxicity Test Showed a Morphological Disruption and Oxidative Stress to Airway Organoids after 1-NP Exposure

We assessed the impact of 1-NP on airway organoids as a proof-of-concept to demonstrate the application of organoids in environmental toxicology. To investigate the impact of 1-NP on airway organoids, we evaluated cell viability using a fluorometric live/dead cytotoxicity assay at two time points after subjecting the airway organoids to different environmentally relevant dosages (1 and 10 μM) and a higher dosage (50 μM). Other commercially available 3D-adapted viability assays, include Alamar blue,^31^ Promega CellTiter-Glo 3D cell viability assay,^32^ and MTT cell viability assay.^33^ However, the readings for all of the wells are processed in bulk, determined by the average viability signal intensity measured across each entire well. Therefore, these approaches neglect the visualization of viability throughout the full cross-sectional area of 3D organoids and the morphological changes that occur in individual organoids following treatment. Consequently, they provide an inadequate representation of the precise live-to-dead cell ratio within each organoid sphere, limiting the depth of insight into the mechanistic effects of pollutants. To address this issue and gain a more comprehensive understanding, our study employs an image-based fluorometric live/dead cytotoxicity assay to assess the viability of 3D cellular models.

**Figure 2:**
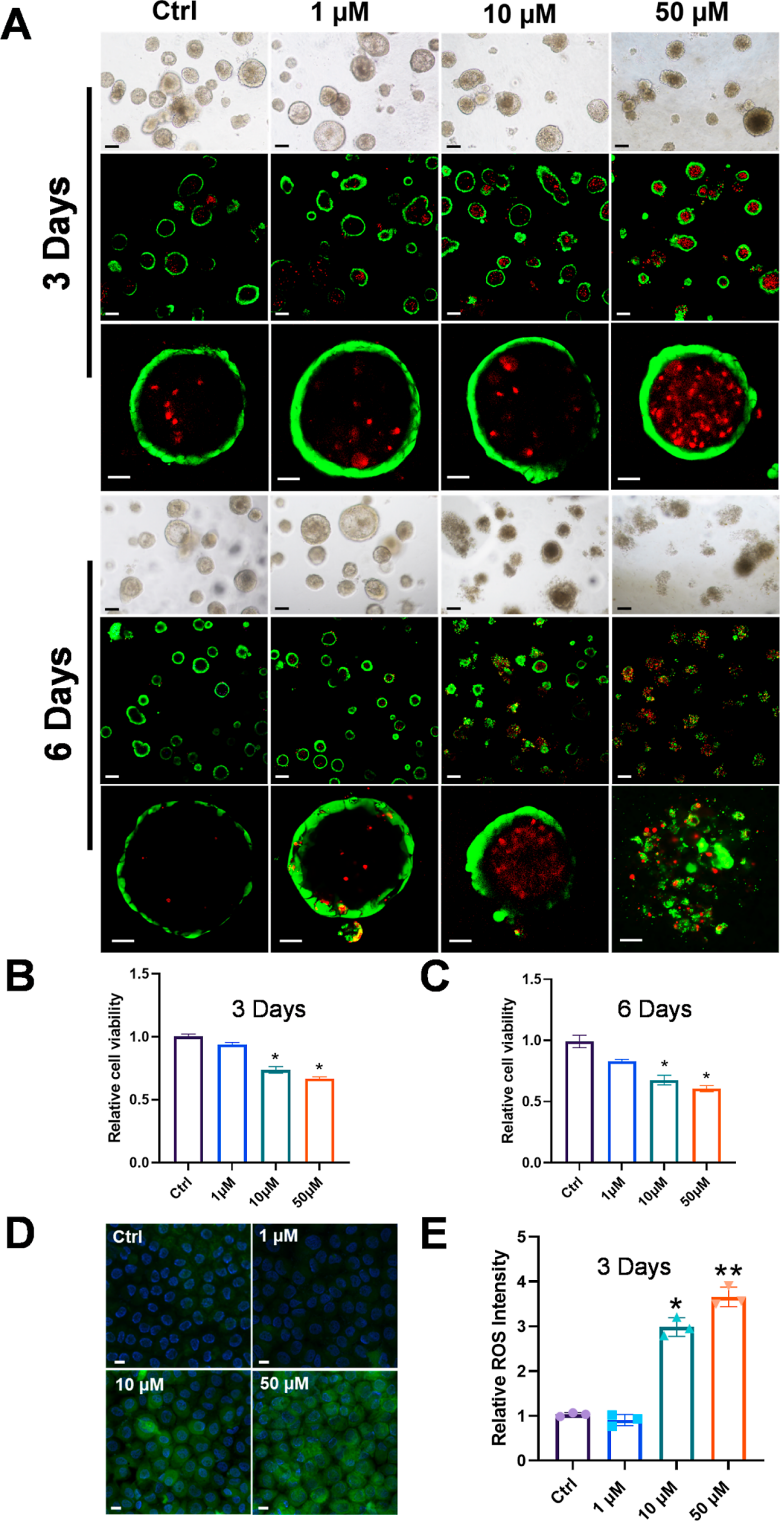
Architecture alteration and visual assessment of cytotoxicity effect on human airway organoids induced by 1-NP treatment. (A) Representative photomicrographs and live/dead staining images of the control group and 1, 10, and 50 μM 1-NP groups at two time points (day 3 and day 6). Green fluorescence indicates the Calcein-AM stain in the live cells and red fluorescence indicates the PI stain in the dead cells. Scale bars, 100 μm. (B,C) Relative cell viability based on fluorometric live/dead cytotoxicity test of 1-NP-treated organoids after exposure for three days (B) and six days (C). (D) Fluorescence microscopic ROS analysis utilized DCFDA (green) in 1-NP (3 days)-treated 2D airway organoids. Scale bars, 10 μm. (E) ROS fluorescence intensity is depicted as a fold change compared to the control 2D airway organoids. All the data are shown as the mean ± standard deviation of *n* = 3, *p < 0.05, **P < 0.01.

Based on both morphological examination and live/dead assays, no obvious morphological change or cytotoxicity was observed under low-dose exposure until day six (Figure 2A–C and Figure S1). At a middle-dose (10 μM) exposure, significant cell toxicity was observed on day 3 (cell viability of 74%) and intensified on day 6 (cell viability of 68%), with obvious morphological alteration (Figure 2A–C). On day 3, for the high-dose exposure group, significant cytotoxicity was observed (cell viability of 67%), and massive dead cells were identified within the lumens of most organoids, while a small portion of organoids completely lost their architecture. On day 6, 39% of cell death was observed, and almost every individual organoid lost integrity (Figure 2A).

Previous cell viability tests using 1-NP treatments on common lung epithelial cell lines revealed that A549 cells had a LC50 of 2.8 μM after 48 h and that BEAS-2B cells had a cell viability of 62% at a dose of 10 μM after 24 h.^23,34^ It is not surprising to see that our model is relatively resistant to 1-NP treatments (cell viability of 74% at 10 μM after 72 h of exposure). Although the potential interlab variation can contribute to these differences, another possibility is that, indeed, its LC50 is relatively high. We speculate that this might be due to the mucus constituting a liquid barrier and diluted 1-NP concentration in contact with epithelial cells, while basal cells at the bottom of the pseudostratified epithelium could differentiate to replenish the dead cells and repair the airway epithelium.^35^ Furthermore, mucociliary clearance afforded by ciliated and goblet cells can further improve the tolerance to pollutants. It was previously reported that PSC-derived alveolar type 2 epithelial cells exhibited a significant decrease in viability at BaP concentrations ≥ 100 nM, indicating higher sensitivity to chemical treatment compared to cancer cell lines.^17^ This vulnerability could be attributed to the fetal-like characteristics inherent in PSC-derived organoids as fetuses are protected by the maternal–fetal barrier and therefore are less resilient to direct exposure to environmental toxins than adults. Therefore, our observation of ASC-derived human airway organoids was likely to represent the airway epithelial response to 1-NP under physiological conditions.

It was known that 1-NP induced a rapid ROS surge and oxidative damage in lung epithelial cell lines,^23^ which was one of the major mechanisms of its cytotoxicity.^23,36^ A significant ROS surge was not induced in airway organoids after treatment with 1 μM 1-NP, whereas significantly increased ROS production was observed in 10 and 50 μM-doses under a 3 day exposure, in agreement with the cytotoxicity assay (Figure 2D,E). However, a substantial increase in ROS was seen at 1 μM after 24 h in A549 cells and at the same concentration but within 2 h in BEAS-2B cells.^23^ Collectively, the toxicity evaluation of 1-NP demonstrated a higher tolerance in human airway organoids compared to common cell lines in both cytotoxicity and ROS assays.

### ROS- and Disease-Associated Lipidome Change in Airway Organoids upon 1-NP Treatment

**Figure 3:**
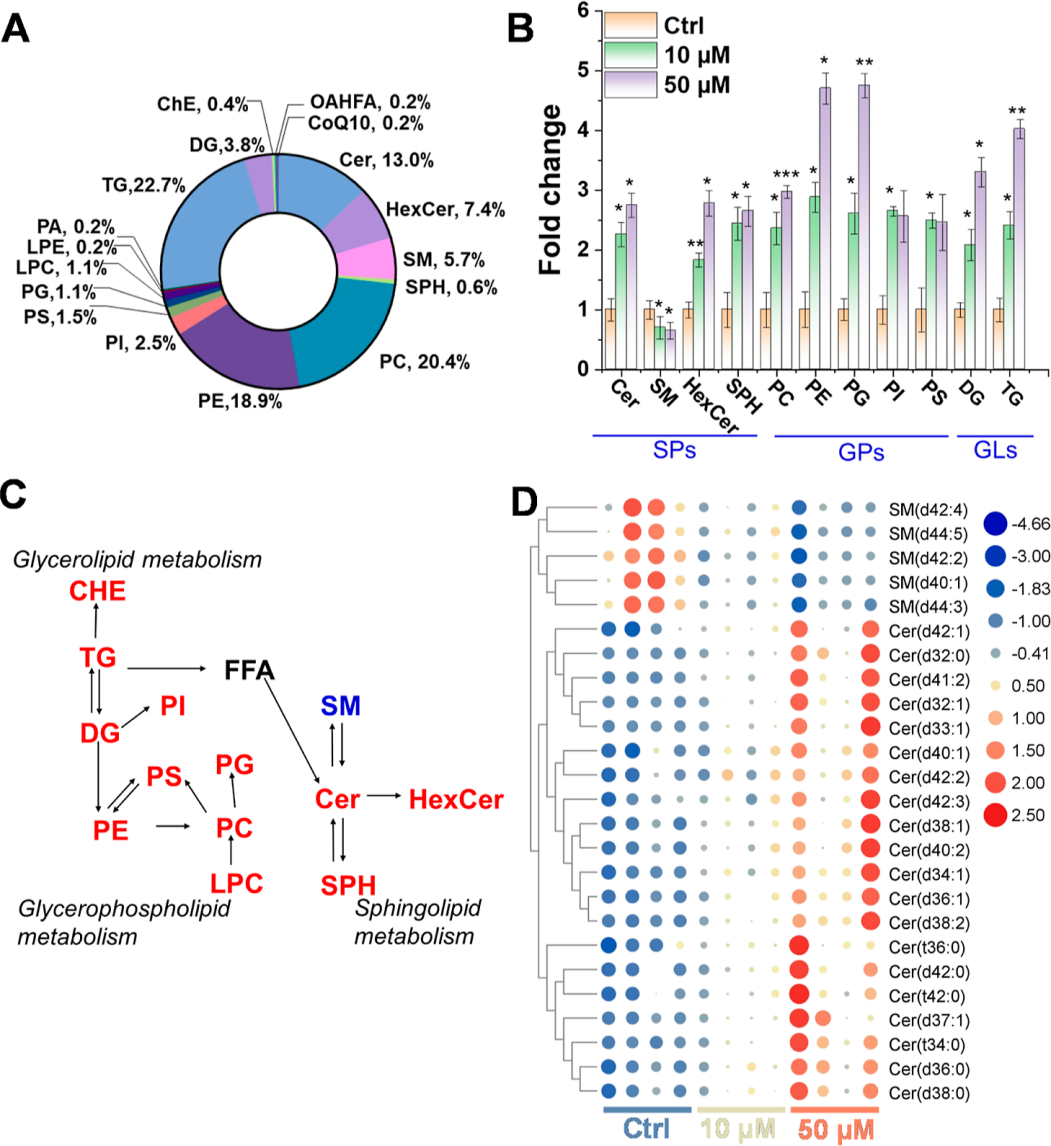
Lipidomic analysis of 3D airway organoids in the control, 10 μM, and 50 μM 1-NP treatment groups. (A) Donut plot showing total lipid identifications and their proportion (%) in organoids. (B) Intensity fold changes of altered lipid classes. Data are the mean ± SD of *n* = 4, *P < 0.05, **P < 0.01, ***P < 0.001 (in comparison to the control group via Student’s *t*-test). (C) 1-NP induced disruption of glycerolipid, glycerophospholipid, and sphingolipid metabolism. Up- and down-regulated lipids were marked in red and blue, respectively. And the lipid with no significant changes was represented in black. (D) Heatmap analysis of the disturbed pattern of SM and Cer lipids.

Based on the aforementioned findings, 10 and 50 μM 1-NP treatments were explored using a global lipidomic study. 471 lipid species from 6 lipid categories and 18 lipid subclasses were identified in all samples (Figure 3A). According to Figure 3A, glycerophospholipids (GP) accounted for the bulk of lipid species (45.7%), followed by glycerolipids (GL) and sphingolipids (SP) at 26.5 and 26.7%, respectively. The principal component analysis (PCA) results revealed distinct patterns between the control and exposed groups (Figure S2), indicating that 1-NP disrupted lipid metabolism. Furthermore, a clear dose-dependent effect could be noticed (Figure S2).

Using the threshold of fold change (FC) > 2 or 0.5 and *p* < 0.05, 112 lipid molecules with consistent regulation patterns under both exposure groups (10 and 50 μM) were identified (Table S3). The lipid classes, including sphingolipids, glycerophospholipids, and glycerolipids, exhibited significant dysregulation, as evidenced by the FC comparison (Figure 3B) and heatmap analysis (Figure S3). This suggests that exposure to 1-NP can cause disruptions in the metabolism of sphingolipids, glycerophospholipids, and glycerolipids in airway organoids (Figure 3C). Among the lipids regulated by 1-NP, the sphingomyelin (SM) lipid class showed a discernible decrease postexposure. Consistent with our ROS data, the accumulation of triglycerides (TG) and diglycerides (DG) was reported to be associated with excessive cellular ROS.^37^ Cer has been reported to induce oxidative stress and trigger apoptotic cell death.^38^ Notably, significant and reciprocal regulation in the opposite direction was observed between SM and Cer lipids, suggesting that 1-NP might promote the conversion from SM to Cer in airway organoids (Figure 3D). Considering that SM conversion to Cer has been linked to apoptosis activation,^28^ we speculated that 1-NP could exert its cytotoxicity by promoting the SM-Cer conversion.

### Mass Spectrometry Imaging Validated Morphological and Molecular Perturbation Induced by 1-NP

In this study, we used MALDI-MSI as another imaging technique for assessing spatial lipid changes resulting from 1-NP treatment. We selected a three-day exposure to 10 μM 1-NP, as guided by previous findings. In a manner comparable to that of the LC–MS data (Figure S2), the pLSA score plots of MSI data exhibited clear distinctions between the control and 1-NP-treated groups in both positive and negative modes (Figure S4), indicating a noticeable disruption in lipid levels post-1-NP exposure.

**Figure 4:**
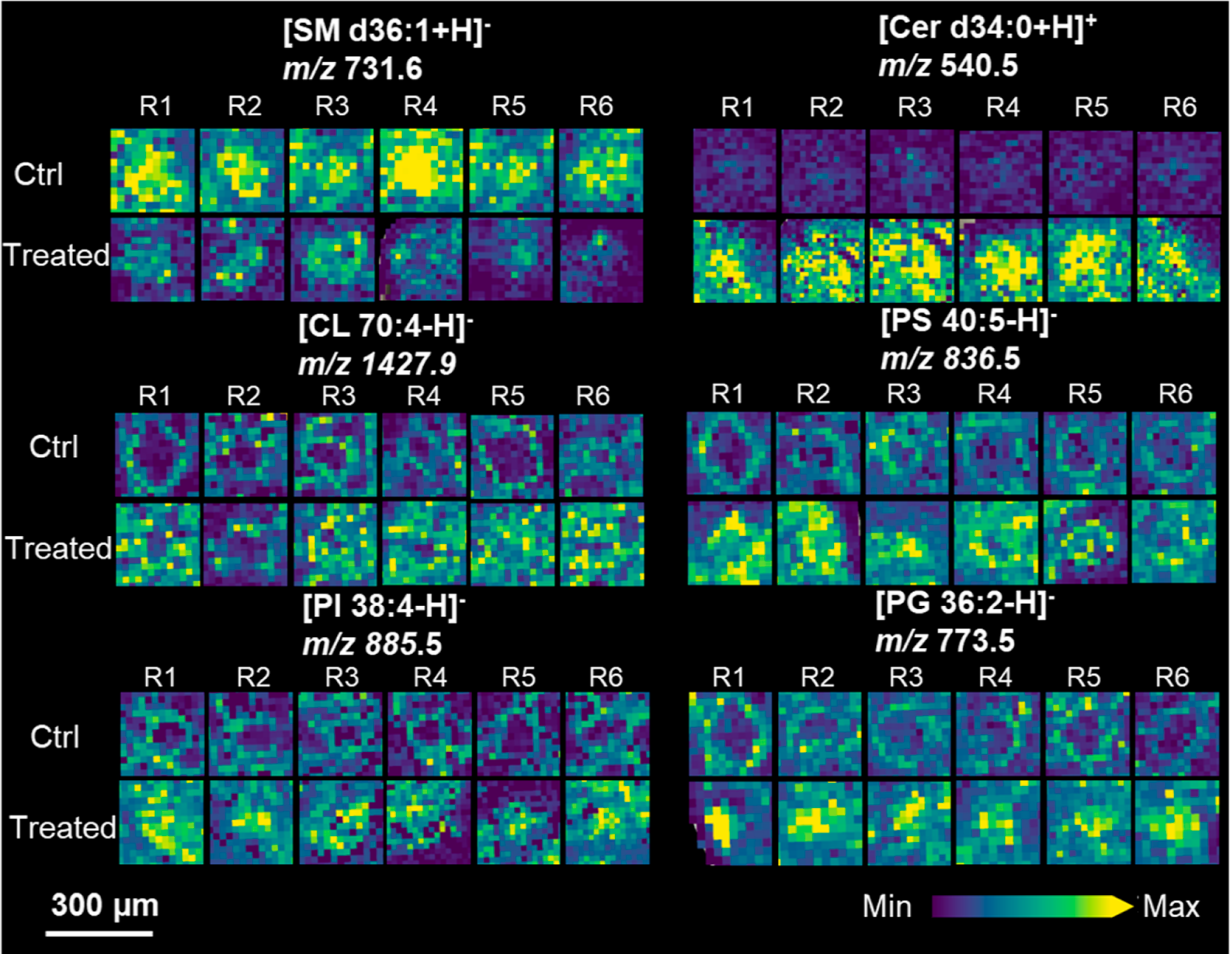
Represented MALDI-MSI images of airway organoids sections were shown in both the positive and negative modes. For each lipid, the upper one represents the control group, while the lower one represents the treated group. Each group consisted of six replicates. Relative ion intensity is indicated by the Viridis color scale bar located at the lower right corner. Scale bar at the lower left corner represents 300 μm.

Lipid species were presumptively identified by comparing LC lipidomic data and performing a search in the Lipid Maps (https://www.lipidmaps.org/) database with a mass tolerance of 5 ppm. Nine differentially expressed lipid species were identified (Table S5) in the MSI spectrum, including glycerophospholipids and sphingolipids. Representative ion images of each differentially expressed lipid subclass are shown in Figure 4, which shows a regulatory pattern in agreement with our lipidomics analysis. SM and Cer (both sphingolipids) were distributed in the whole cellular area. Upon 1-NP exposure, SM underwent downregulation, while Cer experienced upregulation. The conversion of SM to Cer observed in the MSI results is consistent with the lipidomic observations mentioned earlier. The glycerophospholipids marked the external walls of organoids in the control group but dispersed in the exposure group, which was probably due to the accumulation of dead cells in the interior and the collapsed structure of dead organoids. Notably, MSI detected CL, a signature phospholipid of mitochondria.^39^ A three-day 1-NP exposure significantly increased CL abundance. CL was known to be crucial for energy metabolism, and an aberrant level of CL was associated with lung disease onset.^40^ Observations from experimental pneumonia studies and clinical data from human patients indicate a significant increase in the level of CL levels. This rise is possibly due to a malfunctioning transport system within the lungs, causing abnormal buildup in the lung tissue and fluids, which worsens lung injury.^40^ In addition to disrupted transport, impaired enzyme functions in CL metabolism also contributes to elevated CL concentrations. Experiments with zebrafish embryos exposed to 11 environmental chemicals demonstrated increased CL levels, linked to modifications in the enzymes responsible for CL processing.^41^ Imbalances in CL metabolism have detrimental effects, altering anticardiolipin antibody levels, disturbing the thromboxane A2-prostacyclin equilibrium, and promoting clotting incidents, potentially leading to cardiac complications and arrhythmias.^41^ Our observation suggested that 1-NP exposure upregulates CL levels by impacting both the transport process and the CL metabolic enzymes. As essential components for mitochondrial and cellular functionality, disturbances in CL metabolism give rise to detrimental phenotypes. Additionally, increased CL would cause structural damage to pulmonary surfactant membranes.^42^ To the best of our knowledge, it was first discovered that 1-NP could regulate the abundance of CL. The combinatory use of MSI and lipidomics therefore showed complementary effects in revealing detailed molecular information in response to environmental toxins. Additionally, MSI spatially resolves the lipid composition and could be used to characterize the structural features of organoids at the molecular level.

Through the application of physiologically relevant human airway organoids, the toxicity of 1-NP on the native airway epithelium was comprehensively characterized by multimodal imaging and lipidomic analysis. Current PSC-derived organoids resemble fetal tissue and are intricate to generate. In contrast, other existing ASC-derived organoids show limited characterization and lack analysis of cilia movement and cell type quantification.^17−19^ Different from the existing human airway organoids, our ASC-derived organoids are advancing in terms of ease of generation, near-perfect derivation efficiency, robustness, highly mature degree, and stable expansion for over one year. Interestingly, a higher tolerance to pollutants was observed (Figures 2B–E). The cytotoxicity induced by 1-NP may originate from enhancement of the SM-Cer conversion, as revealed by lipidomic profiling. Moreover, multimodal imaging tools, including the cutting-edge MSI technique, were used together as a comprehensive characterization tool for lipidomic analysis. MSI data revealed CL perturbations implicating mitochondrial disruption, which might be caused by the overproduction of ROS. The observation of CL abnormalities therefore implied a potential mechanism for the cytotoxic effects of 1-NP. We believe that this study demonstrates the reliability and strength of ASC-derived airway organoids in investigating the molecular mechanisms of environmental pollutants. Combining the use of advanced MS techniques such as MSI, we could obtain a more detailed, physiologically relevant understanding of the effects of air pollutants on human airways, which consequently sheds light on future environmental health studies.

Our organoid-based toxicity assessment platform indeed holds promise in environmental toxicology but comes with several inherent constraints. First, one key limitation of the airway organoids lies in their inability to reproduce adaptive immunity due to the absence of immune cells within them. Consequently, these organoids fall short of mimicking the roles played by B and T cells, which are pivotal components of the adaptive immune response. The platform primarily reflects the innate immune response through the activity of innate immune receptors, such as TLRs, expressed on epithelial cells. When selecting models to study adaptive immunity-related responses, this limitation must be taken into consideration, given that adaptive immunity involves antigen-specific recognition and expansion of B and T cells following pathogen exposure. To overcome this gap, future work could include a coculture system to include immune cells such as macrophages with advanced 3D organoid systems; Second, our study’s focus was confined to the overall response regarding small molecules, including lipidomics and ROS generation. However, we did not monitor the effects on macromolecules, such as proteins. To obtain a more comprehensive understanding of the toxicological mechanisms of environmental pollutants, the utilization of advanced proteomics analysis techniques such as single-cell proteomics and post-translational modification analysis may be considered. Third, the capability of high-throughput toxicant screening on our platform is constrained due to the manual and time-intensive transfer of organoids from culture plates to multiwell plates with flat-bottom glass surfaces suitable for high-resolution confocal imaging. This challenge could be alleviated by adopting 2D airway organoids in 96-well transwell plates and streamlining image analysis through the application of machine-learning- and deep-learning-powered algorithms, ultimately improving the speed and precision of toxicity assessments. These strategies enable a more in-depth exploration of molecular mechanisms in subsequent investigations.

## Supporting Information Available

Beating cilia in the 3D airway organoids (MP4)
Beating cilia in the 2D airway organoids (MP4)
LC–MS/MS conditions for lipidomics; list of antibodies used in immunofluorescence staining and flow cytometry; M5 sprayer parameters; representative bright-field images of 1, 10, and 50 μM 1-NP groups for 2 days exposure in 3D airway organoids; PCA score plots of lipidomic data from QC samples, DMSO controls, and 10 and 50 μM 1-NP-treated groups; heat map analysis of dysregulated lipids in 3D airway organoids under different dose 1-NP exposure by lipidomic analysis; pLSA score plots of MALDI-MSI profiles obtained from the control and treatment groups; individual lipid species with significant changes of the 1-NP-treated group in comparison with the control group filtered by *p* < 0.05 and FC > 2 or < 0.8 (*n* = 4); and assigned differentially expressed lipids in both positive and negative modes (PDF)
