# Supplementary material for: Human Airway Organoids and Multimodal Imaging-Based Toxicity Evaluation of 1-Nitropyrene
Source: Environ Sci Technol. 2024 Mar 28;58(14):6083–92. doi: 10.1021/acs.est.3c07195 (PMC11008236; doi:10.1021/acs.est.3c07195)
Supplement: Supplementary file 3 — es3c07195_si_003.pdf [file es3c07195_si_003.pdf]

Supporting Information for

Human Airway Organoids and Multimodal Imaging-based Toxicity Evaluation of 1-Nitropyrene

Yingyan Zhou[1], Cun Li[2], Yanyan Chen[1], Yifei Yu[2], Xin Diao[1], Raymond Chiu[2], Jiacheng Fang[1], Yuting Shen[1], Jianing Wang[1], Lin Zhu[1,*], Jie Zhou[2,*], Zongwei Cai[1,*]

[1]State Key Laboratory of Environmental and Biological Analysis, Hong Kong Baptist University, Hong Kong, China

[2]Department of Microbiology, School of Clinical Medicine, Li Ka Shing Faculty of Medicine, The University of Hong Kong, Hong Kong, China

*Corresponding author

E-mail addresses: zwcai@hkbu.edu.hk (Dr. Zongwei Cai); jiezhou@hku.hk (Dr. Jie Zhou); zhu_lin@hkbu.edu.hk (Dr. Lin Zhu).

Number of pages: 10

Number of tables: 4

Number of figures: 4

Contents:

Text S1. LC-MS/MS conditions for lipidomics

Tables

Table S1. List of antibodies used in immunofluorescence staining and flow cytometry

Table S2. M5 spayer parameters

Table S3. Individual lipid species with significant changes of the 1-NP-treated group in comparison with the control group filtered by P<0.05 and fold change (FC) >2 or <0.8 (n=4).

Table S4. Assigned differentially expressed lipids in both positive and negative modes

Figures

Figure S1. Representative bright-field images of 1, 10, 50 μM 1-NP groups for 2 days exposure in 3D airway organoids

Figure S2. PCA score plots of lipidomic data from quality control (QC) samples, DMSO controls, and 10 and 50 μM 1-NP treated groups in both positive (A) and negative modes (B)

Figure S3. Heat map analysis of dysregulated lipids in 3D airway organoids under different dose 1-NP exposure by lipidomics analysis (n=4)

Figure S4. pLSA score plots of MALDI-MSI profiles obtained from the control and treatment groups in both positive (A) and negative mode (B)

Text S1. LC-MS/MS conditions for lipidomics.

The mobile phases for lipidomic analysis consisted of buffer A (10 mM ammonium formate in the ACN/water (60:40 v/v) with 0.1% formic acid) and B (10 mM ammonium formate in the IPA/ACN (90:10 v/v) with 0.1% formic acid). The gradient elution (23 min) at a flow rate of 0.26 mL/min was set as follow: 0.0-2.0 min from 30% B to 45% B, 2.0-7.0 min to 70% B, 7.0-9.0 min to 85% B, 9.0-17.0 min to 100% B and kept for 2.0 min, 19.0-20 min to 30% B and kept for 3.0 min. The injection volume was 10 μL. QC samples were injected at the beginning, end, and after every 4 samples of the run.

The parameters of MS were set as follows: ion transfer tube temperature: 285°C; vaporizer temperature: 300°C; sheath gas flow rate: 50 arbitrary unit; aux gas flow rate: 15 arbitrary unit; spray voltage: 3.0 kV in the positive and negative ionization mode. The resolution was set as 120,000 and the m/z scan range was 100-1200. A positive/negative data-dependent (dd) high-energy collision dissociation (HCD)-MS^2 mode was used for data acquisition.

Table S1. List of antibodies used in immunofluorescence staining and flow cytometry.

Reagents	Company	Catalog No.
Mouse Anti-Cytokeratin 5	Abcam	ab128190
Mouse Anti-β-tubulin 4	Sigma	T7941
Mouse Anti-Mucin 5AC	Abcam	ab3649
Rat Anti-Uteroglobin/CC-10	R&D Systems	MAB4218-SP
Goat Anti-Mouse, Alexa Fluor 488	Invitrogen	A11001
Goat Anti-Rabbit, Alexa Fluor 488	Invitrogen	A11034

Goat Anti-Rat, Alexa Fluor 488	Invitrogen	A-11006

Table S2. M5 spayer parameters.

Matrix	Temperature (℃)	solvent	#Passes	Concentration (mg/mL)	Flow rate (mL/min)	Velocity (mm/min)	Spacing (mm)	Pattern	Drying (s)
DHB	55	100% MeOH	20	20	0.03	0.03	2.5	CC	25
NEDC	65	90% MeOH	15	7	0.05	1800	2	CC	10

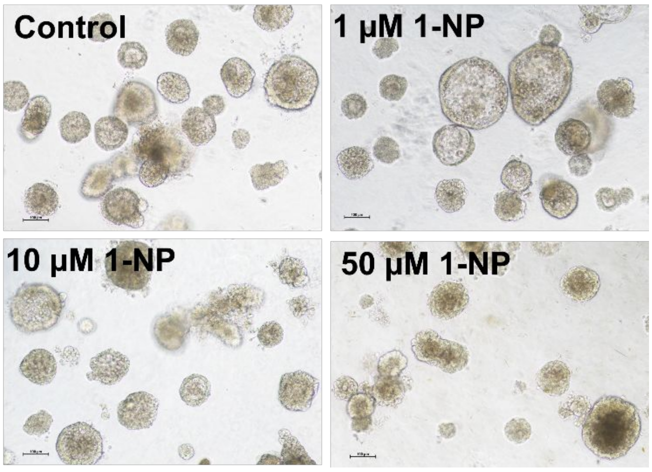

Figure S1. Representative bright-field images of 1, 10, 50 μM 1-NP groups for 2 days exposure in 3D airway organoids. Scale bar, 100 μm.

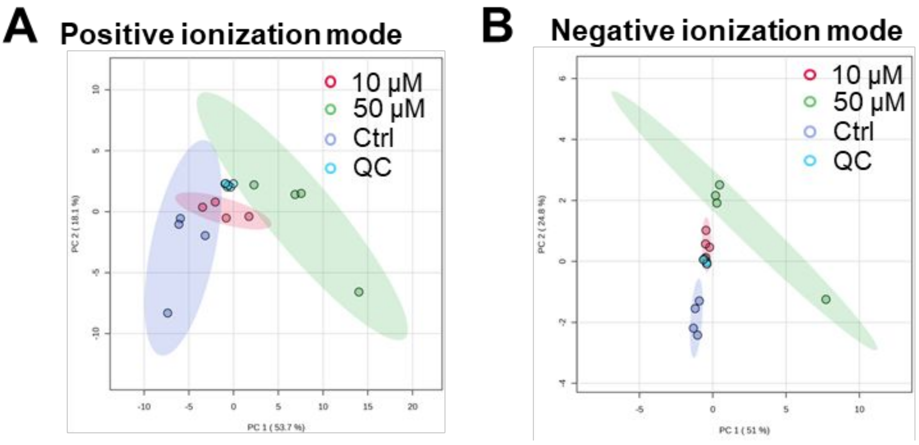

Figure S2. PCA score plots of lipidomic data from quality control (QC) samples,

DMSO controls, and 10 and 50 μM 1-NP treated groups in both positive (A) and negative modes (B).

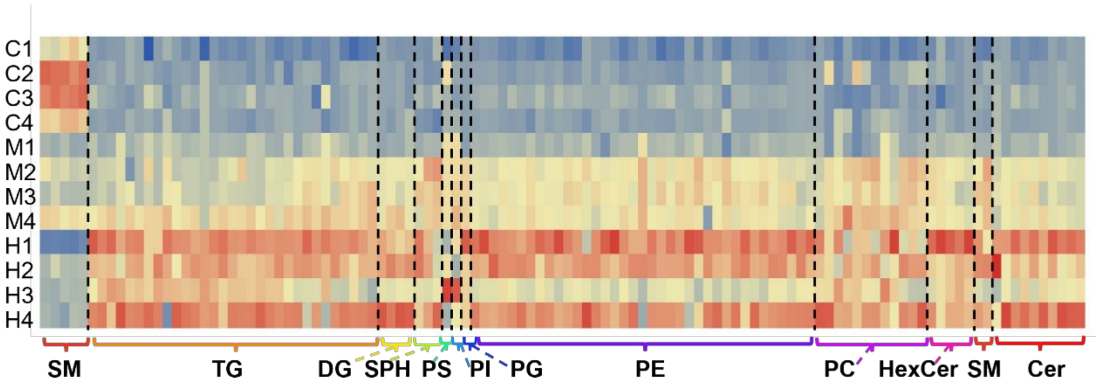

Figure S3. Heat map analysis of dysregulated lipids in 3D airway organoids under different dose 1-NP exposure by lipidomics analysis (n=4). “C”, “M” and “H” represent the control, 10 μM- and 50 μM-dose exposure group, respectively.

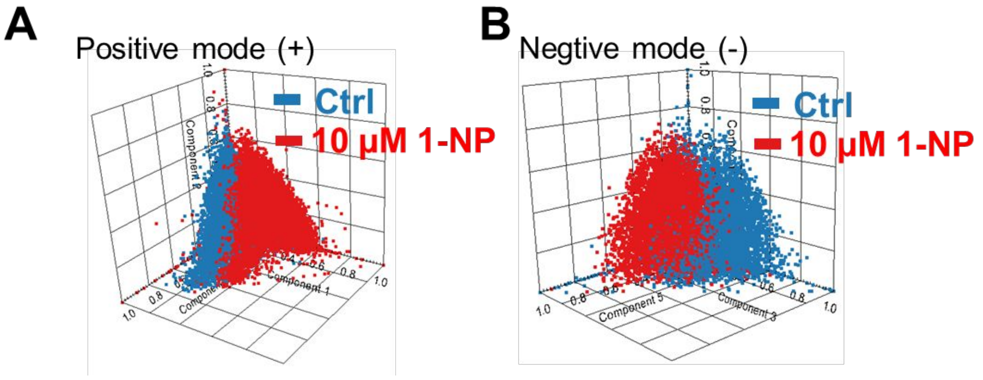

Figure S4. pLSA score plots of MALDI-MSI profiles obtained from the control and treatment groups in both positive (A) and negative mode (B).

Table S3. Individual lipid species with significant changes of the 1-NP-treated group in comparison with the control group filtered by P<0.05 and fold change (FC) >2 or <0.8 (n=4).

Name	FC (10 μM)	*p* value (10 μM)	FC (50 μM)	*p* value (50 μM)
Cer (d18:1/14:0)	2.85	0.018	6.89	0.028

Cer (d17:1/16:0)	2.35	0.029	4.80	0.036
Cer (t18:0/16:0)	2.17	0.0055	3.99	0.0099
Cer (d18:1/16:0)	2.23	0.0068	3.61	0.019
Cer (d20:0/16:0)	2.62	0.010	4.22	0.0038
Cer (d18:1/18:0)	2.06	0.0063	3.13	0.0073
Cer (d38:0)	2.16	0.015	3.59	0.0086
Cer (d18:1/20:1)	2.47	0.012	4.19	0.0094
SM (d32:4)	2.65	0.011	4.00	0.0020
SM (d40:1)	0.59	0.027	0.48	0.0084
SM (d42:2)	0.57	0.0051	0.47	0.00093
SM (d18:1/24:3)	0.64	0.095	0.46	0.021
SM (d44:3)	0.53	0.0077	0.34	0.0016
SM (d44:5)	0.66	0.098	0.44	0.0103
Hex1Cer (d18:0/16:0)	2.48	0.0021	5.62	0.019
Hex1Cer (d18:1/18:0)	2.17	0.00035	3.49	4.5E-05
Hex1Cer (t18:0/16:0)	2.25	0.0010	3.56	0.0091
Hex2Cer (d18:1/16:0)	2.08	0.053	3.61	0.023
Hex3Cer (d18:1/16:0)	2.30	0.0031	3.20	0.019
PC (16:0/16:0)	2.69	0.028	3.43	0.038
PC (16:0/16:1)	2.51	0.026	2.96	0.0089
PC (16:0/18:1)	2.59	0.038	3.34	0.029
PC (36:1)	2.77	0.025	3.41	0.039
PC (16:0/20:4)	2.32	0.027	3.50	0.057
PC (16:1e/20:4)	2.44	0.0020	3.02	0.0059
PC (20:1/18:1)	5.43	0.024	6.12	0.0091
PC (38:3e)	2.45	0.099	1.82	0.043
PC (38:5)	2.09	0.044	2.55	0.0016

PC (20:1/20:2)	2.39	0.076	2.19	0.028
PC (18:3e/22:2)	2.33	0.034	2.64	0.0126
PC (18:1/22:5)	2.13	0.0079	2.73	0.01045
PC (40:8e)	2.64	0.041	4.03	0.00082
PE (18:1e)	5.41	0.011	19.96	0.022
PE (12:0p/16:0)	4.17	0.0028	9.58	0.016
PE (16:0/14:0)	2.48	0.0028	5.98	0.0056
PE (12:0p/18:1)	3.10	0.010	5.64	0.021
PE (16:0/16:0)	3.36	0.0086	6.63	0.0057
PE (16:0p/16:0)	3.72	0.0042	6.84	0.00053
PE (16:0/16:1)	2.16	0.0035	2.89	0.021
PE (16:0p/16:1)	2.83	0.0057	4.82	0.021
PE (18:0/16:0)	2.66	0.020	4.42	0.024
PE (16:0/18:1)	4.03	0.013	7.92	0.022
PE (16:1/18:1)	2.97	0.10	4.39	0.0069
PE (18:1p/16:1)	2.62	0.010	4.60	0.036
PE (16:1e/18:1)	3.18	0.24	10.32	0.017
PE (16:1/18:2)	2.11	0.0032	2.32	0.031
PE (14:0p/20:4)	3.00	0.0038	4.27	0.081
PE (18:0/18:1)	2.79	0.013	4.55	0.0092
PE (16:0p/20:1)	2.11	0.0080	3.99	0.0029
PE (18:1/18:1)	2.66	0.011	4.24	0.0033
PE (36:2e)	2.81	0.0047	5.41	0.0017
PE (16:0p/20:3)	2.72	0.0047	4.81	0.019
PE (16:0p/20:4)	2.90	0.0067	5.12	0.0095
PE (16:0p/20:5)	2.11	0.017	3.62	0.057
PE (16:0p/22:1)	2.78	0.13	6.44	0.022

PE (38:2)	3.96	0.052	8.08	0.015
PE (38:2e)	4.35	0.0033	6.75	0.051
PE (18:0/20:4)	2.76	0.010	4.85	0.0016
PE (18:1/20:4)	2.52	0.0054	3.73	0.0026
PE (18:1p/20:5)	2.04	0.020	3.38	0.043
PE (16:0p/22:6)	2.96	0.0035	5.58	0.014
PE (18:1/22:1)	2.54	0.032	4.63	0.0060
PE (18:0p/22:3)	2.69	0.0024	3.70	0.0022
PE (18:1/22:4)	3.74	0.0065	7.19	0.016
PE (18:0p/22:5)	2.50	0.011	4.45	0.00097
PE (18:1/22:5)	2.80	0.0024	4.85	0.00025
PE (18:0p/22:6)	2.78	0.0096	6.13	0.016
PE (18:1/22:6)	3.15	0.0095	7.12	0.028
PE (18:1p/22:6)	3.02	0.0042	5.95	0.018
PG (16:1/18:1)	2.61	0.049	4.74	0.0049
PG (18:1/18:2)	0.54	0.038	0.41	0.014
PI (16:0/16:0)	2.65	0.00011	2.56	0.088
PS (18:0/22:3)	2.49	0.018	2.45	0.11
SPH (t16:0)	2.20	0.022	2.13	0.063
SPH (t18:0)	4.98	0.021	5.85	0.00062
SPH (t20:0)	3.23	0.017	5.45	0.0032
DG (18:0/18:0)	2.02	0.011	3.59	0.014
DG (18:0/18:1)	2.03	0.060	3.23	0.019
DG (18:1/18:1)	2.11	0.037	3.26	0.015
TG (16:0/14:0/14:0)	2.20	0.011	3.07	0.022
TG (16:0/14:0/16:0)	2.24	0.0081	2.50	0.0076
TG (24:2/10:1/12:1)	2.77	0.35	3.99	0.044

TG (16:0/14:0/18:1)	2.29	0.0048	2.84	0.0056
TG (15:0/16:0/18:1)	2.16	0.0085	3.11	0.0019
TG (16:0/16:1/18:1)	2.22	0.0062	2.98	0.0071
TG (16:0/17:1/18:1)	2.07	0.0063	3.14	0.0021
TG (16:1/17:1/18:2)	4.74	0.01079	6.65	0.0098
TG (16:0/18:1/18:1)	3.21	0.0026	4.52	0.0016
TG (16:1/18:1/18:1)	2.19	0.0073	2.67	0.022
TG (16:1/18:1/18:2)	2.19	0.018	3.65	0.0014
TG (16:1/16:1/20:4)	2.03	0.066	4.18	3.9E-05
TG (18:1/17:1/18:1)	3.35	0.0095	3.97	0.080
TG (16:0e/18:1/20:2)	2.00	0.049	2.40	0.027
TG (18:1/18:1/18:2)	2.27	0.0079	3.71	0.0016
TG (16:1/18:1/20:4)	3.10	0.0059	6.53	0.0017
TG (16:1/16:1/22:5)	2.33	0.061	5.68	4.8E-06
TG (18:1/17:1/20:3)	2.31	0.22	6.05	0.00017
TG (18:1/17:1/20:4)	2.52	0.063	5.63	0.013
TG (18:1/18:1/20:2)	2.31	0.014	4.29	0.00024
TG (16:1/18:1/22:5)	2.87	0.094	6.22	0.0041
TG (19:1/18:1/20:3)	2.56	0.0074	4.93	0.00024
TG (18:1/17:1/22:4)	2.90	0.0076	5.73	0.0028
TG (20:1/18:1/20:2)	2.08	0.018	3.14	0.00022
TG (18:1/18:1/22:3)	2.60	0.0059	4.85	7.8E-05
TG (18:1/18:1/22:4)	2.40	0.039	5.31	0.00022
TG (18:1/18:1/22:5)	3.47	0.011	6.41	0.00091
TG (18:1/18:2/22:5)	3.03	0.049	7.43	2.5E-06
TG (18:1/18:2/23:1)	2.01	0.012	3.42	0.00012
TG (18:1/20:2/22:3)	2.49	0.012	4.94	2.9E-05

TG (18:1/20:2/22:5)	3.25	0.015	7.62	7.2E-05
TG (18:1/20:3/22:5)	4.02	0.041	10.40	0.0090

Table S4. Assigned differentially expressed lipids in both positive and negative modes.

Experimental m/z	Theoretical m/z	Assignment	Adduct	Mass error (ppm)
731.6031	731.6061	SM d36:1	$[M+H]^+$	4.10
540.5346	540.535	Cer 34:0;O2	$[M+H]^+$	0.74
760.5139	760.5134	PS 34:1	$[M-H]^-$	0.66
733.5338	733.5338	PG 36:2	$[M-H]^-$	0
833.5172	833.5186	PI 34:2	$[M-H]^-$	1.68
835.5325	835.5342	PI 34:1	$[M-H]^-$	2.03
885.5503	885.5499	PI 38:4	$[M-H]^-$	0.45
1425.979	1425.981	CL 70:5	$[M-H]^-$	1.12
1427.991	1427.996	CL 70:4	[M-H]-	3.71
